# Monitoring auditory cortical plasticity in hearing aid users with long latency auditory evoked potentials: a longitudinal study

**DOI:** 10.6061/clinics/2018/e51

**Published:** 2018-02-12

**Authors:** Renata Aparecida Leite, Fernanda Cristina Leite Magliaro, Jeziela Cristina Raimundo, Ricardo Ferreira Bento, Carla Gentile Matas

**Affiliations:** I Departamento de Fisioterapia, Fonoaudiologia e Terapia Ocupacional, Faculdade de Medicina (FMUSP), Universidade de Sao Paulo, Sao Paulo, SP, BR; II Departamento de Oftalmologia e Otorrinolaringologia, Faculdade de Medicina (FMUSP), Universidade de Sao Paulo, Sao Paulo, SP, BR

**Keywords:** Auditory Evoked Potentials, Hearing Aid, Child, Neuronal Plasticity

## Abstract

**OBJECTIVE:**

The objective of this study was to compare long-latency auditory evoked potentials before and after hearing aid fittings in children with sensorineural hearing loss compared with age-matched children with normal hearing.

**METHODS:**

Thirty-two subjects of both genders aged 7 to 12 years participated in this study and were divided into two groups as follows: 14 children with normal hearing were assigned to the control group (mean age 9 years and 8 months), and 18 children with mild to moderate symmetrical bilateral sensorineural hearing loss were assigned to the study group (mean age 9 years and 2 months). The children underwent tympanometry, pure tone and speech audiometry and long-latency auditory evoked potential testing with speech and tone burst stimuli. The groups were assessed at three time points.

**RESULTS:**

The study group had a lower percentage of positive responses, lower P1-N1 and P2-N2 amplitudes (speech and tone burst), and increased latencies for the P1 and P300 components following the tone burst stimuli. They also showed improvements in long-latency auditory evoked potentials (with regard to both the amplitude and presence of responses) after hearing aid use.

**CONCLUSIONS:**

Alterations in the central auditory pathways can be identified using P1-N1 and P2-N2 amplitude components, and the presence of these components increases after a short period of auditory stimulation (hearing aid use). These findings emphasize the importance of using these amplitude components to monitor the neuroplasticity of the central auditory nervous system in hearing aid users.

## INTRODUCTION

Hearing thresholds obtained using behavioral tests do not provide comprehensive data regarding the contribution of individual sound amplification devices (hearing aids [HAs]) and/or cochlear implants to the central auditory nervous system. For this reason, the use of objective tests is essential 1.

Currently, long-latency auditory evoked potentials (LLAEPs) are used to investigate impairments in the central auditory pathways of children 1,2 and adults 3,4 with sensorineural hearing loss (SNHL). LLAEPs are also used as a biomarker for changes in the cortical auditory pathway after the use of HAs or cochlear implants 1-3,5.

LLAEPs are composed of the P1, N1, P2, N2 and P300 components. P1, N1 and P2 are considered exogenous components, i.e., they are influenced by the acoustic characteristics of a stimulus, while the N2 and P300 components are endogenous, i.e., they are most influenced by intrinsic events such as perception and cognition 6.

LLAEPs can be obtained using different acoustic stimuli (pure tones or speech), and responses to tonal stimuli have lower latency than those obtained from a speech stimulus 7.

Impairment of the LLAEP components (P1, N1, P2, N2) in children with mild to moderate SNHL has been reported in a previous study, which showed that this population has a deficit in central auditory processing 8. In addition, the changes to the LLAEP components indicate improved responses from auditory stimulation; for example, the P1 component shows a decrease in latency after the use of a cochlear implant 1,2.

According to the literature, the presence of the P1, N1, P2 and N2 components using speech and tone burst stimuli in children with SNHL is positively correlated with the duration of HA use, and the latency of the N1 and P2 components is positively correlated with both the duration of HA use and the child’s age 9.

The P300 component is present among people with hearing loss as long as the individual can perceive and distinguish acoustic stimuli 10. A study showed that individuals with normal hearing or mild SNHL exhibited 100% P300 presence at intensities of 65 dB SPL and 80 dB SPL, subjects with moderate SNHL showed 50% P300 presence at an intensity of 65 dB SPL and 100% P300 presence at an intensity of 80 dB SPL, and individuals with severe/profound SNHL showed 14.3% P300 presence at an intensity of 65 dB SPL and 11.1% P300 presence at an intensity of 80 dB SPL. In addition, individuals who had P300 present at a lower intensity (65 dB SPL) showed increased latency, decreased amplitude and impaired morphology of the P300 component 4.

Individuals (children and adults) with severe or profound congenital hearing loss showed 58.6% presence of P300. Individuals with profound hearing loss showed lower amplitudes than those with severe hearing loss, but there were no differences in latency 11.

Given the importance of proper central auditory nervous system functioning in childhood development, the aim of this study was to provide scientific evidence for the development and plasticity of the central auditory nervous system in response to HA use using LLAEPs. The study will evaluate the effectiveness and benefits of sound amplification (stimulation) in hearing-impaired children. This study compares LLAEPs in children with SNHL before and after HA fittings compared with those in age-matched children with normal hearing.

## METHODS

This was a longitudinal prospective clinical study approved by the Ethics Committee for Research Project Analysis under Protocol No 266512/2013. The participants’ legal guardians read and signed the Terms of Free and Informed Consent (TFIC) form, and the children signed the Terms of Assent form.

A total of 32 subjects of both genders aged 7 to 12 years participated in this study. The participants were divided into two groups as follows: 14 comprised the control group (CG; mean age 9 years and 8 months), and 18 comprised the study group (SG; mean age 9 years and 2 months).

The CG comprised children who had normal otoscopy findings, auditory thresholds up to 15 dB HL (frequencies of 250 to 8000 Hz), a type A tympanometric curve 12, ipsilateral acoustic reflexes (frequency 500-4000 Hz) and no auditory or language complaints or neurological impairments.

The SG comprised children who had normal otoscopy findings, mild to moderate bilateral symmetrical and flat SNHL 13 with a speech recognition threshold (words) without amplification between 72 and 100%, no prior use of any type of sound amplification device, a type A tympanometric curve 12 and no neurological impairments.

Hearing loss in the SG was diagnosed at the Hearing Clinic of the Clinical Hospital of the School Medicine of the University of São Paulo - Division of Clinical Otorhinolaryngology (Ambulatório de Saúde Auditiva do Hospital das Clínicas da Faculdade de Medicina da Universidade de São Paulo – Divisão de Clínica da Otorrinolaringologia do HCFMUSP*)*. All children with hearing loss were referred to the Auditory Health Clinic of the Clinics Hospital by the Central System for Regularization of Health Services Offering (Sistema Central de Regularização de Oferta de Serviços de Saúde – Cross) of the State Health Department of São Paulo. Due to the above referral system, at the time of the audiological and electrophysiological hearing assessments, none of the children had undergone any type of rehabilitation for their hearing loss.

The etiology of the hearing loss was unknown in 83.34% (N=15), suspected prematurity in 5.55% 1, possible neonatal anoxia in 5.55% 1 and suspected genetic but with no further investigation in 5.55% of the children.

After the children were diagnosed with hearing loss, they were referred for electrophysiological evaluation of hearing. At a later date, they returned to the outpatient clinic for selection and fitting of HAs.

In all cases, the HA was a bilateral mini retroauricular-type device with digital technology and non-linear signal processing. All children with mild or moderate hearing loss underwent a HA fitting. The children used their HAs daily, and the daily use was monitored through the processing algorithm that is built into the HA’s memory (mean time of 8.5 hours of daily use).

The children underwent tonal and vocal audiometry using a Grason Stadler GSI 61 audiometer and a TDH 50 supra-aural earphone, acoustic emittance measurements using a Madsen Model Zodiac 901 emittance meter, and LLAEP examination using a two-channel device (the Smart Box Jr.™ Smart EP universal model, Intelligent Hearing Systems) calibrated at listening level (dBHL). The acoustic stimulation used to acquire the LLAEPs was presented by a sound field system with speakers positioned at a 90-degree angle and 45 cm from the ear to be tested.

Both groups were assessed at three different time points: baseline (M0), 3 months after the initial assessment (M3), and 9 months after the initial assessment (M9). The SG children did not use HAs at M0.

The LLAEPs were obtained by the same evaluator at the three time points. The components were analyzed by the evaluator and two other professionals with experience in hearing electrophysiology using consensus scoring.

To capture the P1, N1, P2, N2 and P300 components of the LLAEPs, the child was placed in a reclining chair in an acoustically treated room with appropriate electrical access. The skin was cleaned with abrasive paste, and the electrodes were attached to the skin with electrolytic paste and adhesive tape (Micropore) at the active vertex (Cz) and ground (Fpz) based on the International Electrode System standard IES 10-20 14. The reference electrodes were positioned on the left and right mastoids (M1 and M2). Responses were collected when the impedance values were below 5 kohms. Band-pass filters of 1 to 30 Hz were used with a 150 K gain, rejection above 66.7 microvolts (µV), and speech and tone-burst stimuli.

LLAEPs were evoked by tone burst stimuli using 1000 Hz as the frequent stimulus and 2000 Hz as the rare stimulus. The stimuli were delivered for 50 ms at 75 dBnHL at a display speed of 1.1 stimuli per second using an exact Blackman envelope and an interstimulus interval (ISI) of 860 ms. We chose the frequencies between 1000 and 2000 Hz based on the literature and used the protocol proposed by Hall, 2007.

**Figure 1:**
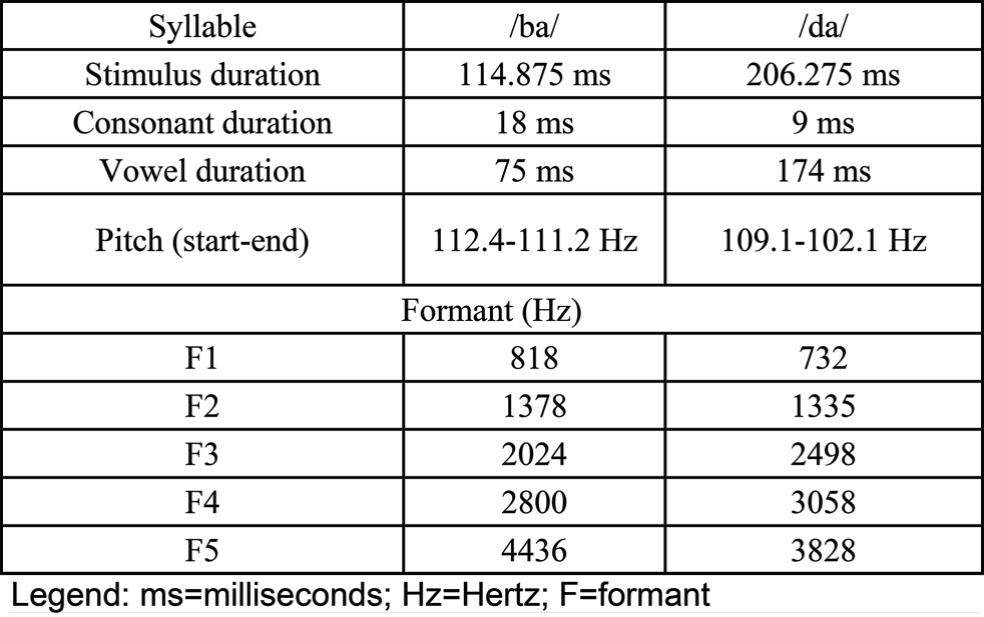
Specific characteristics of the /ba/ and /da/ speech stimuli used to obtain the LLAEPs.

For the speech stimuli, the /ba/ syllable was used as the frequent stimulus and the /da/ syllable was used as the rare stimulus. The stimuli were presented at 75 dBnHL at a presentation rate of 1.1 stimuli per second. The frequent stimulus (/ba/) had a duration of 114 ms and an ISI of 799 ms, while the rare stimulus (/da/) had a duration of 206 ms and an ISI of 690 ms. Both stimuli were synthetic. The specific characteristics of the /ba/ and /da/ speech sounds are described in Figure 1.

A total of 300 stimuli (15% rare and 85% frequent) were used to capture the P300 component. The children were instructed to pay attention to the rare stimuli, which occurred randomly among a series of frequent stimuli, and to raise their hand whenever the rare event occurred.

During data collection, responses were recorded on two charts: one chart corresponded to the frequent stimulus and identified and analyzed the P1, N1, P2 and N2 components, and another chart corresponded to the rare stimulus and identified and analyzed the P300 component. The amplitude and latency of all components and the presence and absence of responses for each ear were analyzed.

Each child was instructed to look at a fixed point two meters in front of him/her. The charts were accepted for analysis when a maximum of 30 artifacts were present.

In addition to the tabulation of the latency and amplitude values of the LLAEP components at each assessment point, the LLAEP components were classified as present or absent for each individual and for each studied ear.

### Statistical methods

First, the percentages of present and absent LLAEP components were calculated for both groups. We compared the latency and amplitude values within each group and between groups at each time point (M0, M3 and M9).

The Shapiro-Wilk and Levene tests were performed to verify that the groups had a normal distribution and to determine whether there was homogeneity of variances between groups, respectively.

Descriptive analyses were performed using means and standard deviations (± SD). When comparing three values, the three-factor repeated measures analysis of variance was used. When comparing two values, the two-factor analysis or the Bonferroni test was used. The significance level was set at 5% (*p* value ≤0.05) for all analyses.

## RESULTS

**Figure 2:**
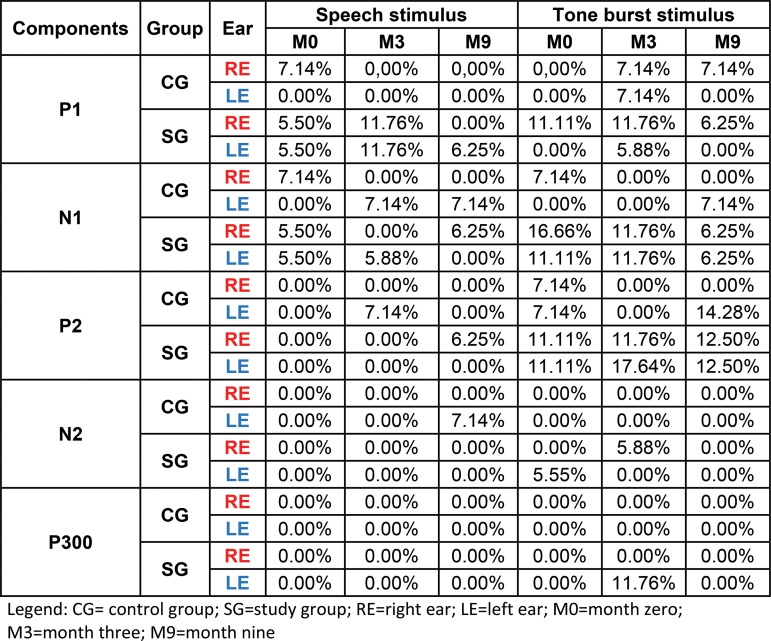
Percentages of LLAEP component absences in the study and control groups.

In this study, low percentages of non-responses were observed for both speech stimulus and tone bursts in both ears for both the SG and CG at all time points (Figure 2).

The results of the statistical analyses, including the latency and amplitude values of all the components, are presented below.

### LLAEPs with speech stimuli

#### P1, N1, P2, N2 and P300 Amplitudes

**Figure 3:**
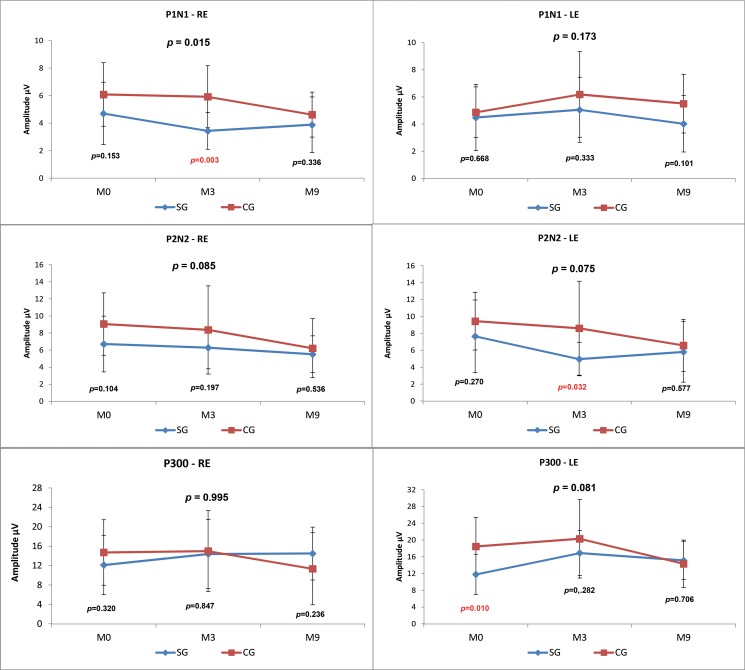
Comparison of the LLAEP component amplitudes evoked by speech stimuli between the CG and SG at the three time points.

When the CG and SG were compared at the three time points (M0, M3, M9), there was a statistically significant difference (*p*-value=0.015) in the P1-N1 amplitude value in the right ear (RE), and the SG presented a lower mean amplitude at all assessment points (Figure 3).

We observed lower amplitudes at all time points in the SG compared with the CG; however, this result was only statistically significant for P1 in the RE at M3 (*p*=0.003), P2-N2 in the LE at M3 (*p*=0.032) and P300 at M0 (*p*=0.010) (Figure 3).

**Figure 4:**
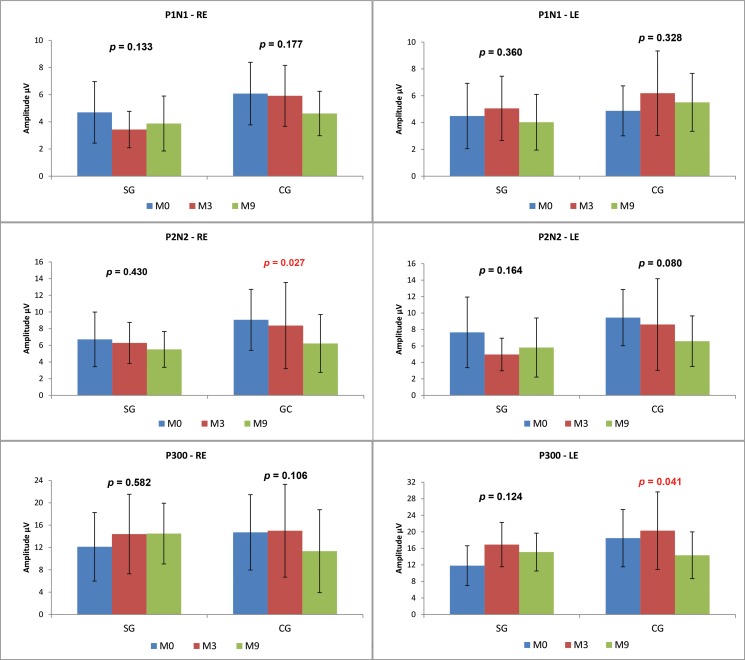
Comparison of the LLAEP component amplitudes evoked by speech stimuli across the three time points between the CG and SG.

Comparisons between the time points revealed no statistically significant differences in the SG. In the CG, there was a statistically significant decrease over time in the P2-N2 amplitude in the RE (M0xM3xM9 *p*=0.027; M0xM9 *p*=0.060) and in the P300 amplitude in the LE (M0xM3xM9 *p*=0.041; M3xM9 *p*=0.056) (Figure 4).

#### P1, N1, P2, N2 and P300 component latency

The comparison of the CG and SG at the three time points and the two-factor comparison revealed no statistically significant difference in the latency of the LLAEP components (Table 1).

When comparing the latency between time points, statistically significant decreases in P2 (M0xM3xM9 *p*=0.007; M3XM9 *p*=0.010) and N2 (M0xM3xM9 *p*=0.005; M3xM9 *p*=0.007) were found for the LE in the SG. In the CG, a statistically significant increase in the P2 latency (M0xM3xM9 *p*=0.005; M0xM3 *p*=0.005) in the LE was observed (Table 2).

### LLAEP with tone burst stimulus

#### P1, N1, P2, N2 and P300 amplitudes

**Figure 5:**
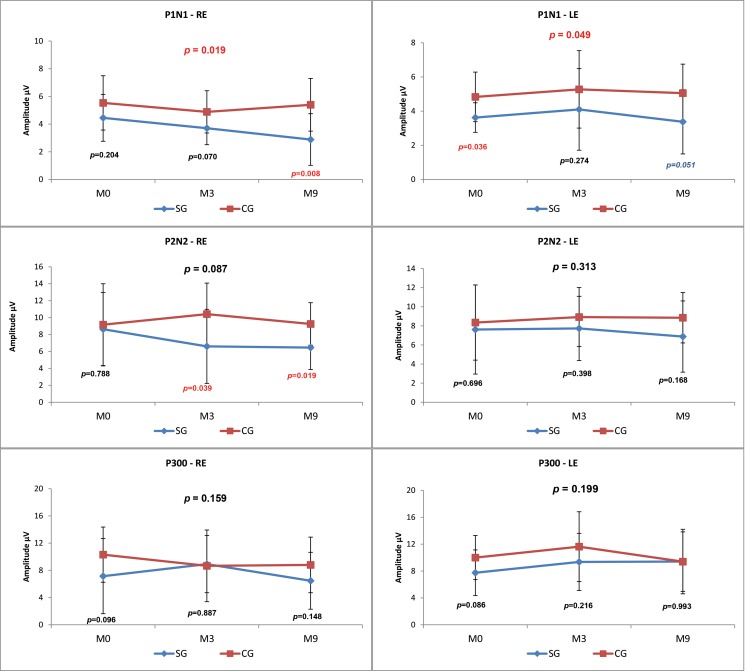
Comparison of the LLAEP component amplitudes evoked by tone burst stimuli between the CG and SG at the three time points.

When comparing the LLAEP components between the CG and SG at the three time points (M0, M3 and M9), statistically significant differences in the P1-N1 amplitude were found in both ears, and the SG had lower amplitudes at all time points (Figure 5).

When the results of CG and SG were compared at the different time points, we found a significant difference in the amplitude of P1-N1 in the RE at M9 (*p*=0.008) and in the LE at M1 (*p*=0.036). We also observed significant differences in P2-N2 in the RE at M3 (*p*=0.039) and M9 (*p*=0.019) (Figure 5). For both of the above amplitudes, we found lower values in the SG.

**Figure 6:**
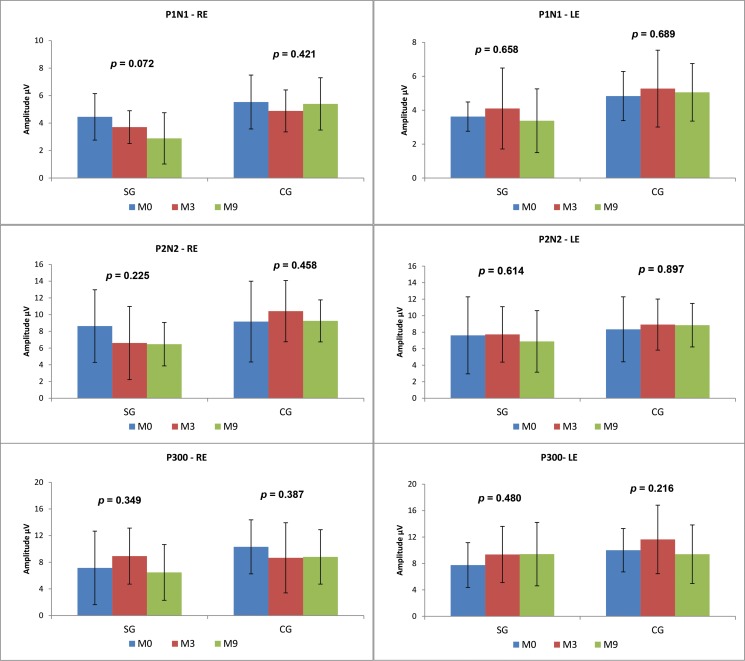
Comparison of the LLAEP component amplitudes evoked by tone burst stimuli across the three time points for the CG and SG.

Comparisons between the time points revealed no statistically significant differences between the P1-N1, P2, N2 and P300 amplitudes in either the CG or SG (Figure 6).

#### P1, N1, P2, N2 and P300 component latency

The comparison of the control and study groups at the three time points revealed no significant difference in the latency of the LLAEP components. In the two-factor comparison, we observed a significant difference only for P300 at M0 (*p*=0.013) in the LE, with the SG showing a greater latency value.

In the comparison of the three time points within each group, we found significant differences only for P1 of the LE in the CG (M0xM3xM9 *p*=0.032; M0xM3 *p*=0.043; M3xM9 *p*=0.048) and P1 in the LE (M0xM3xM9 *p*=0.023; M0xM9 *p*=0.002) and P300 in the RE (M0xM3xM9 *p*=0.019; M0xM3 *p*=0.013) in the SG (Table 4).

## DISCUSSION

Currently, LLAEPs are used to investigate central auditory pathway impairments in children 1,2 and adults 3,4 with SNHL. They are also used as a biomarker for changes in the cortical auditory pathway after initiating HA use or receiving a cochlear implant 1,2,3,5.

Regarding the presence or absence of P1, N1, P2, N2 and P300 component responses in the SG and CG, there was a low percentage of absent responses in both groups for speech and tone burst stimuli. However, the SG had a greater percentage of absent responses than the CG at all time points, especially in response to tone burst stimuli (Figure 2). Therefore, we hypothesize that these findings may be related to the complexity of the stimuli used. Because a speech stimulus is more acoustically complex than a tone burst, more structures or neurons may be activated, leading to a more apparent electrophysiological response.

The P1 and N2 components are predominant in the LLAEPs of small children. Between 3 and 6 years old, the P2 component appears and the N2 becomes more clear and robust 15. The N1 component is not present in small children and can only be observed with the use of a long ISI (greater than 1 second) in children between 3 and 10 years old 16. It should be noted that, according to the literature, the higher the ISI (e.g., 800 ms), the greater the likelihood that the N1 and P2 components will be present starting at 8 years old 17,18. In the present study, the CG and SG presented low percentages of non-response for all components; therefore, the inter-stimulus range used in this study (799 ms for speech and 860 ms for tone burst) was adequate for eliciting responses in the studied age group.

When the two groups were compared, the N1 and P2 components in the SG showed a greater percentage of non-responses to tone burst stimuli (Figure 2). This finding suggests that children with mild to moderate SNHL have a maturational delay in the cortical auditory pathway caused by auditory deprivation. This finding is consistent with the reports in the literature 8 that the N1 and P2 components are poorly defined in children with mild to moderate SNHL.

Another important finding was that only the N1 component obtained with the tone burst stimuli showed a decrease in non-response in both ears during HA use, reinforcing the idea that this population has a maturational delay in the auditory pathway and that stimulation with an HA facilitates the maturational process and the emergence of the N1 component.

According to the literature, the presence of the P1, N1, P2 and N2 components evoked by both speech and tone burst stimuli is positively correlated with the duration of HA use 9. Thus, we hypothesize that the P2 component may emerge after a longer duration of stimulation.

We observed that the P1-N1 and P2-N2 amplitudes (Figures 3 and 5) in response to both the speech and tone burst stimuli were lower in the SG than in the CG at all time points and in both ears. These findings indicate that the children in the SG showed less neuronal activation in the regions generating these components even after the use of HAs, consistent with reports that hearing deficiency impairs normal development of the connectivity necessary for the formation of a functional auditory system 2.

Furthermore, it is known that amplitudes of the P1, N1 and P2 (exogenous) components are influenced by the physical characteristics of the stimulus 19,20. We speculate that the lower amplitudes presented by the SG individuals with mild to moderate hearing loss occurred because they perceived the stimulus (75 dBnHL) at a lower intensity (dB NS).

The comparison of the P300 amplitudes in both the CG and SG (Figures 3 and 5) revealed a significant difference in response to the speech stimulus at M0 (LE), where the SG had a lower amplitude; in contrast, at M3 and M9, the mean P300 amplitudes ?were similar between the groups. It should be noted that P300 was present in almost 100% of the subjects (Figure 2). The similarity of these values in the two groups and the presence of this component in most individuals may be explained by the task used to obtain the P300 amplitude. As the emergence of P300 is affected by the cognitive activity performed (endogenous potential) 6 more so than the characteristics of the stimulus 19, we can hypothesize that mild to moderate hearing loss did not directly impair the activation of the cortical structures responsible for performing the task.

These findings are similar to those reported in a study showing that P300 was present in 100% of subjects with normal hearing or mild to moderate SNHL at an intensity of 80 dB SPL 4.

Furthermore, although the difference was not statistically significant, the SG showed an improvement in the P300 amplitude between M0 and M3 for both stimulus types. These results suggest possible neuroplasticity due to HA use. According to the literature, neuroplasticity occurs as a result of the nervous system’s ability to reorganize its structures, functions and connections in response to intrinsic and extrinsic stimuli 21. Therefore, we believe that the improvement in the sound input due to the use of the HA was responsible for the increase in the P300 amplitude observed in response to the stimuli.

According to the literature 2, for children up to the age of 3.5 years with congenital hearing impairment who are stimulated (via cochlear implant use), the P1 latency reaches normal values between 3 and 6 months after cochlear implant activation. In contrast, children who receive cochlear implants between the ages of 3.5 and 7 years have varying responses in terms of latency, and children who receive their implant after age 7 do not have normal P1 latency or wave morphology, even after years of stimulation.

In this study, the comparison of responses to the speech stimulus between the CG and SG (Tables 1 and 3) showed no difference in the mean latency of the P1, N1, P2, N2 and P300 components; however, when the tone burst was used, the SG showed a greater mean latency for P300 in the LE at M0 compared with the CG.

The results of this study show that, for most LLAEP components, the SG and CG had equivalent acoustic stimulus transmission/processing speeds. These findings may be related to the SG’s degree of hearing loss. It is noteworthy that in mild/moderate recruitment hearing loss, the impairment may reside in the outer hair cells (OHCs) 22. Furthermore, stimuli over 40-60 dB directly stimulate the inner hair cells (IHCs) 23, which would be preserved in the population evaluated in this study. Thus, as the LLAEPs were obtained at a high intensity (75 dBHL), the degree of hearing loss did not seem to interfere with the latency times of the SG, which were equivalent to those of the CG.

Another possible cause of the similar latency found in both groups may be related to plasticity resulting from auditory deprivation, i.e., the central nervous system reorganizes to compensate for a peripheral receptor disorder 24. According to the literature, the mechanisms of this compensatory plasticity are not well defined; however, it is known that it can occur due to the following events: activation of previously existing but silent circuits, stabilization of transitional connections that would disappear under normal circumstances, emergence of axons adjacent to injured or inactive regions and various combinations of these events 25. The fact that these data were derived from a population with mild to moderate SNHL, i.e., the acoustic stimulation of the cortical central auditory pathway is reduced but is still stimulated, favors the theory of compensatory plasticity before the child begins to use a HA, which may explain why there were no differences in latency between the SG and CG even after the use of a HA for 9 months.

Although there were no differences in latency between the two groups, the intra-group comparisons at the different time points revealed some differences (Tables 2 and 4). The SG showed improvements in the P2 (M3 X M9) and N2 (M0 x M3, M3 X M9) components in the LE when stimulated with speech sounds. When the tone burst stimulus was used, the SG showed improvements (as a result of the decrease in the latency) in the P1 component in the LE from M0 to M9 and for P300 in the RE from M0 to M3.

The results show that after three months of HA use, there were improvements in the response generation times of the P2 and N2 (speech stimulus) and P1 and P300 (tone burst stimulus) components, likely due to neuroplasticity in response to auditory stimulation (HA use).

These findings are in accordance with a previous study 1. Although the study only investigated P1, the study showed that P1 exhibited decreased latency after HA and/or cochlear implant use, highlighting the importance of P1 as a biological marker of central auditory pathway development in hearing-impaired individuals.

There are some limitations to the current study, such as the number of individuals in the sample. The number of participants in the SG was variable at different time points due to the longitudinal design, which depended on the compliance of patients for audiological follow-up. For this reason, it was not possible to achieve an equal number of individuals at the three time points for audiological follow-up. In addition, it was not possible to obtain a homogeneous sample with consistent etiology and hearing loss onset. Regarding the control group, we believe that the low number of individuals may be related to the fact that they were typical developed children, with no hearing complaints.

In conclusion, the results of this study demonstrated reductions in amplitudes of the P1-N1 and P2-N2 components of the LLAEP and a greater percentage of absent responses in children with mild to moderate SNHL, suggesting a deficit in the activation of the cortical structures responsible for auditory processing of the exogenous characteristics of these stimuli.

P300 showed similar responses between the groups, suggesting that the endogenous potential was less affected than the exogenous potential by the sensory input deficiency caused by the hearing loss in this population.

The latency of the LLAEP components showed little variability between the groups, indicating that sensory hearing loss did not interfere with the stimulus processing speed. The responses were steady across the three time points, demonstrating that this measurement is minimally affected over a short time interval (9 months).

The results of the present study show that it is possible to identify changes in the central auditory pathway using P1-N1 and P2-N2 component amplitudes and that there is an increased response of these components to a short period of auditory stimulation (i.e., HA use). Therefore, the importance of using these components to monitor the neuroplasticity of the central auditory nervous system in HA users should be emphasized.

## AUTHOR CONTRIBUTIONS

Leite RA participated in the conception and design of the study, acquisition, analysis and interpretation of the data, drafting and revision of the manuscript for important intellectual content, and approval of the final version of the manuscript to be published. Magliaro FC participated in the conception and design of the study, acquisition, analysis and interpretation of the data, drafting and revision of the manuscript for important intellectual content, and approval of the final version of the manuscript to be published. Matas CG participated in the conception and design of the study, the analysis and interpretation of the data, drafting and revision of the manuscript for important intellectual content, and approval of the final version of the manuscript to be published. Raimundo JC participated in the conception and design of the study, acquisition of data, and critical revision of the manuscript for important intellectual content. Bento RF participated in critically revising the manuscript for important intellectual content.

## Figures and Tables

**Table 1:** Comparison of the P1, N1, P2, N2 and P300 wave latencies (in ms) obtained from speech stimuli between the control and study groups at the three time points.

Speech	Latencies (ms)								
			Control			Study			*p*-value
			N	Mean	SD	N	Mean	SD	
**P1**	**M0**	**RE**	13	95.4	14.6	13	93.8	9.0	0.749
		**LE**	13	94.4	11.1	13	88.5	18.2	0.333
	**M3**	**RE**	13	89.2	13.5	13	94.6	17.1	0.375
		**LE**	13	91.0	10.0	13	93.9	12.1	0.507
	**M9**	**RE**	13	86.9	13.9	13	95.9	11.7	0.087
		**LE**	13	92.8	5.4	13	95.8	10.1	0.341
**N1**	**M0**	**RE**	12	137.3	11.3	13	139.6	19.0	0.722
		**LE**	12	134.3	14.7	13	134.2	25.0	0.998
	**M3**	**RE**	12	139.8	20.7	13	132.9	20.8	0.414
		**LE**	12	135.8	13.5	13	138.6	19.3	0.683
	**M9**	**RE**	12	132.9	13.6	13	135.1	24.1	0.788
		**LE**	12	137.1	12.9	13	138.0	14.2	0.867
**P2**	**M0**	**RE**	12	184.3	10.2	14	182.6	14.0	0.721
		**LE**	12	179.1	6.6	14	185.9	16.5	0.196
	**M3**	**RE**	12	184.8	14.1	14	184.4	10.6	0.934
		**LE**	12	194.2	11.8	14	195.7	10.4	0.726
	**M9**	**RE**	12	186.0	11.3	14	184.7	13.1	0.793
		**LE**	12	184.1	14.2	14	182.3	18.9	0.789
**N2**	**M0**	**RE**	13	245.2	19.0	15	251.9	15.6	0.314
		**LE**	13	242.1	16.1	15	246.1	14.1	0.490
	**M3**	**RE**	13	252.7	12.8	15	247.0	17.1	0.335
		**LE**	13	251.5	8.1	15	257.1	15.8	0.253
	**M9**	**RE**	13	246.8	16.6	15	257.2	27.3	0.410
		**LE**	13	243.7	12.9	15	242.9	20.8	0.910
**P3**	**M0**	**RE**	14	283.6	29.4	12	284.3	36.1	0.953
		**LE**	14	281.4	21.0	12	295.1	29.4	0.179
	**M3**	**RE**	14	300.1	41.2	12	283.4	30.3	0.257
		**LE**	14	288.9	30.1	12	282.7	24.7	0.576
	**M9**	**RE**	14	290.1	22.4	12	279.8	41.6	0.430
		**LE**	14	278.2	29.4	12	276.8	19.6	0.891

M0 = month zero; M3 = month three; M9 = month nine; RE = right ear; LE = left ear; SD = standard deviation; N = sample size; *p-value considered statistically significant.

**Table 2:** Comparison of the P1, N1, P2, N2 and P300 wave latencies (in ms) obtained from speech stimuli across the three time points for the control and study groups.

Speech	Latencies (ms)									
	Time points	Ears	Control			*p*-value	Study			*p*-value
			N	Mean	SD		N	Mean	SD	
**P1**	**M0**	**RE**	13	95.4	14.6	M0xM3xM9	13	93.8	9.0	M0xM3xM9
		**LE**	13	94.4	11.1	RE=0.857	13	88.5	18.2	RE=0.090
	**M3**	**RE**	13	89.2	13.5	LE=0.193	13	94.6	17.1	LE=0.748
		**LE**	13	91.0	10.0		13	93.9	12.1	
	**M9**	**RE**	13	86.9	13.9		13	95.9	11.7	
		**LE**	13	92.8	5.4		13	95.8	10.1	
**N1**	**M0**	**RE**	12	137.3	11.3	M0xM3xM9	13	139.6	19.0	M0xM3xM9
		**LE**	12	134.3	14.7	RE=0.416	13	134.2	25.0	RE=0.530
	**M3**	**RE**	12	139.8	20.7	LE=0.801	13	132.9	20.8	LE=0.795
		**LE**	12	135.8	13.5		13	138.6	19.3	
	**M9**	**RE**	12	132.9	13.6		13	135.1	24.1	
		**LE**	12	137.1	12.9		13	138.0	14.2	
**P2**	**M0**	**RE**	12	184.3	10.2	M0xM3xM9	14	182.6	14.0	M0xM3xM9
		**LE**	12	179.1	6.6	RE=0.937	14	185.9	16.5	RE=0.823
	**M3**	**RE**	12	184.8	14.1	LE=0.005*	14	184.4	10.6	LE=0.007*
		**LE**	12	194.2	11.8	M0xM3=0.005*	14	195.7	10.4	M3xM9=0.010*
	**M9**	**RE**	12	186.0	11.3		14	184.7	13.1	
		**LE**	12	184.1	14.2		14	182.3	18.9	
**N2**	**M0**	**RE**	13	245.2	19.0	M0xM3xM9	15	251.9	15.6	M0xM3xM9
		**LE**	13	242.1	16.1	RE=0.343	15	246.1	14.1	RE=0.143
	**M3**	**RE**	13	252.7	12.8	LE=0.107	15	247.0	17.1	LE=0.005*
		**LE**	13	251.5	8.1		15	257.1	15.8	M3xM9=0.007*
	**M9**	**RE**	13	246.8	16.6		15	257.2	27.3	
		**LE**	13	243.7	12.9		15	242.9	20.8	
**P3**	**M0**	**RE**	14	283.6	29.4	M0xM3xM9	12	284.3	36.1	M0xM3xM9
		**LE**	14	281.4	21.0	RE=0.262	12	295.1	29.4	RE=0.944
	**M3**	**RE**	14	300.1	41.2	LE=0.383	12	283.4	30.3	LE=0.105
		**LE**	14	288.9	30.1		12	282.7	24.7	
	**M9**	**RE**	14	290.1	22.4		12	279.8	41.6	
		**LE**	14	278.2	29.4		12	276.8	19.6	

M0 = month zero; M3 = month three; M9 = month nine; RE = right ear; LE = left ear; SD = standard deviation; N = sample size; *p-value considered statistically significant.

**Table 3:** Comparison of the P1, N1, P2, N2 and P300 wave latencies (in ms) obtained with tone burst stimuli between the control and study groups at the three time points.

Tone burst	Latencies (ms)								
			Control			Study			*p*-value
			N	Mean	SD	N	Mean	SD	
**P1**	**M0**	**RE**	13	78.70	9.40	13	79.10	18.80	0.946
		**LE**	13	79.60	7.90	13	85.30	18.70	0.932
	**M3**	**RE**	13	72.30	10.50	13	78.40	20.80	0.369
		**LE**	13	71.30	13.20	13	84.90	21.30	0.074
	**M9**	**RE**	13	75.50	8.60	13	75.80	16.60	0.951
		**LE**	13	80.30	6.40	13	77.00	21.80	0.616
**N1**	**M0**	**RE**	12	122.20	20.70	13	79.10	18.80	0.193
		**LE**	12	112.90	13.30	13	85.30	18.70	0.745
	**M3**	**RE**	12	108.20	8.60	13	108.80	14.60	0.893
		**LE**	12	106.10	12.00	13	112.70	21.80	0.369
	**M9**	**RE**	12	109.40	12.70	13	107.30	21.90	0.778
		**LE**	12	109.10	10.00	13	105.60	19.10	0.580
**P2**	**M0**	**RE**	12	169.10	16.90	14	162.00	23.70	0.421
		**LE**	12	169.20	12.70	14	167.20	25.40	0.815
	**M3**	**RE**	12	161.60	13.50	14	162.20	18.60	0.939
		**LE**	12	160.20	17.60	14	161.40	19.60	0.875
	**M9**	**RE**	12	156.90	12.20	14	164.60	19.80	0.282
		**LE**	12	160.20	13.40	14	168.80	21.20	0.264
**N2**	**M0**	**RE**	13	215.20	27.90	15	221.40	16.40	0.479
		**LE**	13	215.80	25.40	15	224.90	21.00	0.308
	**M3**	**RE**	13	223.20	23.90	15	221.60	17.70	0.845
		**LE**	13	214.90	23.80	15	219.90	21.50	0.564
	**M9**	**RE**	13	226.70	11.70	15	226.90	23.10	0.975
		**LE**	13	228.60	23.60	15	219.70	26.40	0.357
**P3**	**M0**	**RE**	14	285.20	31.80	12	309.80	49.00	0.128
		**LE**	14	279.90	25.60	12	319.10	48.80	0.013*
	**M3**	**RE**	14	277.30	34.50	12	277.60	36.30	0.830
		**LE**	14	286.50	35.60	12	294.90	39.80	0.560
	**M9**	**RE**	14	290.90	52.50	12	297.80	62.40	0.753
		**LE**	14	280.80	27.70	12	308.60	63.30	0.144

M0 = month zero; M3 = month three; M9 = month nine ; RE = right ear; LE = left ear; SD = standard deviation; N = sample size; *p-value considered statistically significant.

**Table 4:** Comparison of the P1, N1, P2, N2 and P300 wave latencies (in ms) obtained using tone burst stimuli across the three time points for the control and study groups.

Tone burst	Latencies (ms)									
	Time points	Ears	Control			*p*-value	Study			*p*-value
			N	Mean	SD		N	Mean	SD	
**P1**	**M0**	**RE**	13	78.70	9.40	M0xM3xM9	13	79.10	18.80	M0xM3xM9
		**LE**	13	79.60	7.90	RE=0.157	13	85.30	18.70	RE=0.635
	**M3**	**RE**	13	72.30	10.50	LE=0.032*	13	78.40	20.80	LE=0.023*
		**LE**	13	71.30	13.20	M0xM3=0.043*	13	84.90	21.30	M0xM9=0.0022*
	**M9**	**RE**	13	75.50	8.60	M3xM9=0.048*	13	75.80	16.60	
		**LE**	13	80.30	6.40		13	77.00	21.80	
**N1**	**M0**	**RE**	12	122.20	20.70	M0xM3xM9	13	79.10	18.80	M0xM3xM9
		**LE**	12	112.90	13.30	RE=0.102	13	85.30	18.70	RE=0.941
	**M3**	**RE**	12	108.20	8.60	LE=0.152	13	108.80	14.60	LE=0.444
		**LE**	12	106.10	12.00		13	112.70	21.80	
	**M9**	**RE**	12	109.40	12.70		13	107.30	21.90	
		**LE**	12	109.10	10.00		13	105.60	19.10	
**P2**	**M0**	**RE**	12	169.10	16.90	M0xM3xM9	14	162.00	23.70	M0xM3xM9
		**LE**	12	169.20	12.70	RE=0.181	14	167.20	25.40	RE=0.792
	**M3**	**RE**	12	161.60	13.50	LE=0.150	14	162.20	18.60	LE=0.248
		**LE**	12	160.20	17.60		14	161.40	19.60	
	**M9**	**RE**	12	156.90	12.20		14	164.60	19.80	
		**LE**	12	160.20	13.40		14	168.80	21.20	
**N2**	**M0**	**RE**	13	215.20	27.90	M0xM3xM9	15	221.40	16.40	M0xM3xM9
		**LE**	13	215.80	25.40	RE=0.192	15	224.90	21.00	RE=0.605
	**M3**	**RE**	13	223.20	23.90	LE=0.173	15	221.60	17.70	LE=0.443
		**LE**	13	214.90	23.80		15	219.90	21.50	
	**M9**	**RE**	13	226.70	11.70		15	226.90	23.10	
		**LE**	13	228.60	23.60		15	219.70	26.40	
**P3**	**M0**	**RE**	14	285.20	31.80	M0xM3xM9	12	309.80	49.00	M0xM3xM9
		**LE**	14	279.90	25.60	RE=0.634	12	319.10	48.80	RE=0.019*
	**M3**	**RE**	14	277.30	34.50	LE=0.779	12	277.60	36.30	M0xM3=0.013*
		**LE**	14	286.50	35.60		12	294.90	39.80	LE=0.127
	**M9**	**RE**	14	290.90	52.50		12	297.80	62.40	
		**LE**	14	280.80	27.70		12	308.60	63.30	

M0 = month zero; M3 = month three; M9 = month nine ; RE = right ear; LE = left ear; SD = standard deviation; N = sample size; *p-value considered statistically significant.

## References

[1] Sharma A, Dorman MF, Kral A (2005). The influence of a sensitive period on central auditory development in children with unilateral and bilateral cochlear implants. Hear Res.

[2] Sharma A, Gilley PM, Dorman MF, Baldwin R (2007). Deprivation-induced cortical reorganization in children with cochlear implants. Int J Audiol.

[3] Dawes P, Munro KJ, Kalluri S, Edwards B (2014). Auditory acclimatization and hearing aids: late auditory evoked potentials and speech recognition following unilateral and bilateral amplification. J Acoust Soc Am.

[4] Oates PA, Kurtzberg D, Stapells DR (2002). Effects of sensorineural hearing loss on cortical event-related potential and behavioral measures of speech-sound processing. Ear Hear.

[5] Eggermont JJ, Ponton CW (2003). Auditory – evoked potential studies of cortical maturation in normal hearing and implanted children: correlations with changes in structure and speech perception. Acta Otolaryngol.

[6] McPherson DL (1995). Late Potentials of the Auditory System.

[7] Swink S, Stuart A (2012). Auditory long latency responses to tonal and speech stimuli. J Speech Lang Hear Res.

[8] Koravand A, Jutras B, Lassonde M (2012). Cortical auditory evoked potentials in children with a hearing loss: a pilot study. Int J Pediatr.

[9] Hassaan MR (2011). Aided evoked cortical potential: an objective validation tool for hearing aid benefit. EJENTAS.

[10] Musiek FE, Lee WW, MusieK FE, Rintelmann WF (2001). Potenciais auditivos de média e longa latência. Perspectivas Atuais em Avaliação Auditiva.

[11] Reis AC, Iório MC (2007). [P300 in subjcts with hearing loss]. Pro Fono.

[12] Jerger J (1970). Clinical experience with impedance audiometry. Arch Otolaryngol.

[13] Northen JL, Dows MP (1984). Hearing in children.

[14] Klem GH, Lüders HO, Jasper HH, Elger C (1999). The ten-twenty electrode system of the International Federation. The International Federation of Clinical Neurophysiology. Electroencephalogr Clin Neurophysiol Suppl.

[15] Hall JW, Hall JW (2007). Auditory Late Responses (ALRs). New Handbook of Auditory Evoked Response.

[16] Sharma A, Kraus N, McGee TJ, Nicol TG (1997). Developmental changes in P1 and N1 central auditory responses elicited by consonant-vowel syllables. Electroencephalogr Clin Neurophysiol.

[17] Ceponiene R, Cheou M, Näätänen R (1998). Interstimulus interval and auditory event-related potentials in children: evidence for multiple generators. Electroencephalogr Clin Neurophysiol.

[18] Sussman E, Steinschneider M, Gumenyuk V, Grushko J, Lawson K (2008). The maturation of human evoked brain potentials to sounds presented at different stimulus rates. Hear Res.

[19] Hillyard SA, Picton TW, Hillyard SA, Picton TW (2011). Electrophysiology of Cognition. Comprehensive Physiology.

[20] Prakash H, Abraham A, Rajashekar B, Yerraguntla K (2016). The Effect of Intensity on the Speech Evoked Auditory Late Latency Response in Normal Hearing Individuals. J Int Adv Otol.

[21] Cramer SC, Sur M, Dobkin BH, O’Brien C, Sanger TD, Trojanowski JQ (2011). Harnessing neuroplasticity for clinical applications. Brain.

[22] Moore BCJ, Glasberg BR (1997). A model of loudness perception applied to cochlear hearing loss. Audit Neurosci.

[23] Castro NP, Figueiredo MS, Lopes-Filho O (1997). Audiometria Eletrofisiológica. Tratado de Fonoaudiologia.

[24] Boéchat EM, Fernandes FDM, Mendes BCA, Navas ALPGP (2010). Plasticidade e Amplificação. Tratado de Fonoaudiologia.

[25] Lent R, Lent R (2005). Os neurônios se transformam. Bases Biológicas da Neuroplasticidade. Cem Bilhões de Neurônios. Conceitos Fundamentais de Neurociência.

